# Probenecid Inhibits Extracellular Signal-Regulated Kinase and c-Jun N-Terminal Kinase Mitogen-Activated Protein Kinase Pathways in Regulating Respiratory Syncytial Virus Response

**DOI:** 10.3390/ijms252212452

**Published:** 2024-11-20

**Authors:** Les P. Jones, Harrison C. Bergeron, David E. Martin, Jackelyn Murray, Fred D. Sancilio, Ralph A. Tripp

**Affiliations:** 1 Department of Infectious Diseases, University of Georgia, Athens, GA 30602, USA; lj66@uga.edu (L.P.J.); h.c.bergeron@gmail.com (H.C.B.); jcrab@uga.edu (J.M.); 2 TrippBio, Inc., Jacksonville, FL 32256, USA; davidmartin@trippbio.com; 3 Department of Chemistry and Biochemistry, Florida Atlantic University, Jupiter, FL 33431, USA; fredsancilio@clearwayglobal.com

**Keywords:** antiviral, transcription factor, JNK, ERK, HNF, RSV

## Abstract

We examined the effect of probenecid in regulating the ERK and JNK downstream MAPK pathways affecting respiratory syncytial virus replication. Background: We have previously shown that probenecid inhibits RSV, influenza virus, and SARS-CoV-2 replication in vitro in preclinical animal models and in humans. In a Phase two randomized, placebo-controlled, single-blind, dose range-finding study using probenecid to treat non-hospitalized patients with symptomatic, mild-to-moderate COVID-19, we previously showed that a 1000 mg twice daily treatment for 5 days reduced the median time to viral clearance from 11 to 7 days, and a 500 mg twice daily treatment for 5 days reduced the time to viral clearance from 11 to 9 days more than the placebo. Methods: In this study, we sought to determine the mechanism of action of the probenecid inhibition of RSV replication in human respiratory epithelial (A549) cells. Results: We show that probenecid inhibits the RSV-induced phosphorylation of JNKs and ERKs and the downstream phosphorylation of c-jun, a component of the AP-1 transcription complex needed for virus replication. The inhibition of JNKs by probenecid reversed the repression of transcription factor HNF-4. Conclusion: The probenecid inhibition of JNK and ERK phosphorylation involves the MAPK pathway that precludes virus replication.

## 1. Introduction

Viruses can cause diseases ranging from mild to life-threatening, making effective antiviral drugs an urgent necessity [1]. An ideal antiviral drug is safe, administered orally, cost-effective, and broad-acting [2,3]. Unfortunately, most drug options are not ideal and often lead to drug resistance, are typically specific to a particular virus, and may be expensive, with questionable safety. Viruses are parasites that lack the necessary components for their own replication, and thus depend on the cell machinery of the host to reproduce [4]. This poses a challenge for antiviral drugs, as those that eliminate the virus can also harm the host cells. The purpose of this study was to investigate the mechanism of action (MOA) of probenecid on the mitogen-activated protein kinase (MAPK) pathways involved in virus replication. There are three subfamilies of MAPK, i.e., extracellular signal-regulated kinases (ERKs), c-Jun N-terminal kinases (JNKs), and p38 kinases [5]. MAPK activation results in a series of phosphorylation events in the MAPK cascade, altering mRNA’s translation to proteins. The activation of MAPK signaling controls a diverse cellular response, including the host response to viral infection [6]. ERK activation leads to the phosphorylation of its kinase activity, affecting many downstream targets. Additionally, JNKs are activated by various extracellular stimuli (e.g., proinflammatory cytokines) to phosphorylate various cytoplasmic and nuclear substrates involved in diverse cellular processes [5]. It is known that JNK phosphorylation affects the downstream targets and that MAPK signaling pathways converge in the nuclear translocation of specific factors that promote gene expression, which is required for virus replication. JNK phosphorylation affects organic anion transporters (e.g., OAT3) and transcription factors (TFs), such as hepatocyte nuclear factor 4 (HNF4), and HNF4 has been shown to regulate the expression of various fatty acids [7]. HNF4 also regulates OATs and OAT3 [8]. Previously, we showed that OAT3 is important for influenza virus replication in A549 cells [9]. This finding led us to using probenecid, a chemical inhibitor of OAT3, to inhibit influenza virus replication and the probenecid treatment of RSV and SARS-CoV2 to inhibit replication in vitro and in vivo [10,11,12]. OAT3 is highly conserved and abundantly expressed in epithelial cells [13]. This is notable because JNK activity is affected by viruses [14], probenecid can inhibit JNK phosphorylation [15], and probenecid is a pan-antiviral that also inhibits proinflammatory responses to virus infection [9,10,12,16,17].

Antiviral drugs have been discovered through advancements in our understanding of virus replication. Typically, virus replication begins with the virus attaching to a host cell receptor and fusing with the host cell membrane to enter the cell. Subsequently, the virus releases its nucleic acid into the cell, where virus replication and protein synthesis occur. Finally, different components of the virus are made and assembled to create an infectious virus particle that is released from the host cell [18]. Antiviral drugs that directly target the virus replication pathways include the inhibitors of virus attachment and entry, the inhibitors of virus uncoating, polymerase and protease inhibitors, the inhibitors of nucleoside and nucleotides, and the inhibitors of integrase [19]. Despite the wide range of antiviral drugs investigated, only a few are FDA-approved primarily due to limited efficacy and side effects. Antiviral drugs have two fundamental modes of action—targeting the virus or the host cell. Protease inhibitors are an example of antiviral drugs that target the virus [20]. They inhibit viral assembly by inhibiting the viral proteases that cleave the viral precursor proteins needed for viral replication. Protease inhibitors have been successful in reducing disease and saving lives. One example is antiretroviral therapy (ART) [21]. The process of ART involves using a combination of HIV medicines to lower the viral load to an undetectable level. Many of these antiviral drugs are prodrugs, meaning they require processing or activation before they become effective [22,23]. An example of an antiviral drug targeting a virus is the treatment of COVID-19 with remdesivir, a nucleoside analog RNA-dependent RNA polymerase (RdRP) inhibitor [24]. As viruses replicate quickly, they often develop drug resistance. The higher the virus replication rate is, the higher the mutation rate is, and the more likely drug resistance will develop. Even a single nucleotide change can lead to critical amino acid substitutes in the target protein, resulting in the development of antiviral drug resistance. As a result, there is a need for host-targeted antiviral drugs that are not susceptible to these mutagenic changes and can act broadly against multiple viruses.

The threat of virus infection and disease is high for those who are not vaccinated, emphasizing the need for antiviral drugs [25]. COVID-19 and its many variants further highlight the need for antivirals, particularly those that can act as broad-spectrum drugs. Genome-wide RNAi screens have helped identify the host genes and pathways that viruses use for replication [26,27], leading to drug repurposing for important respiratory viruses like SARS-CoV-2, influenza, and RSV [11,28,29]. Targeting the host cell pathways used for virus replication can be an effective approach for developing antiviral drugs as it targets the essential pathways used by viruses, rather than relying on the discovery of the host genes needed for virus replication [28,29]. Using RNAi genome-wide screens, we found 287 human host cell genes that influenced influenza A virus replication and confirmed that 119 genes inhibited A/WSN/33 (H1N1), and 121 genes inhibited A/Hamburg/04/2009 (H1N1), with 72 of those genes overlapping for the two viruses [29]. The analysis of these validated host genes showed that most genes were linked to host pathways that affected the trafficking of influenza virions to late endosomes early in infection. We validated the RNAi findings using human A549 cells infected with various influenza strains. This allowed us to determine the host genes that affected virus replication based on a virus plaque replication phenotype. Distilling the druggable host genes identified, we showed that OAT3 (SLC22A8) was involved in A/WSN/33 replication [9]. As OAT3 was implicated, we tested probenecid, an FDA-approved drug used for treating gout and hypertension that is non-cytotoxic and specifically inhibits OAT3. Our studies showed that probenecid treatment can reduce the OAT3 mRNA and protein levels in A549 cells and that it can also reduce the influenza A lung titers whether administered in vitro or in a mouse model [9]. Recent studies have shown that probenecid has antiviral effects against SARS-CoV-2, influenza virus, and RSV both in vitro and in vivo [11,12], as well as robust efficacy in humans in a Phase two dose finding clinical study [16]. Probenecid treatment inhibited the replication of SARS-CoV-2 and its variants (Beta, Gamma, Delta, and Omicron, B.1.1) in Vero E6 cells and normal human bronchial epithelial (NHBE) cells at submicromolar concentrations ranging from 0.00001 to 100 μM [12]. In addition, submicromolar concentrations of probenecid inhibited the RSV replication of three RSV strains (A2, A/Memphis-37, and B1 [10]), influenza A virus (A/WSN/33, H1N1, A/New Caledonia/20/99, H1N1, A/California/04/09, H1N1, and A/Philippines/2/82/X-79, H3N2), and the highly pathogenic avian influenza viruses (HPAI) H5N1 and H7N9 [30]. Given the broad antiviral efficacy among virus types, strains, and variants, the antiviral effects of probenecid are thought to involve a shared host cell pathway.

In this study, to avoid biosafety concerns, we studied the MOA of probenecid in RSV-infected A549 cells using comparable ranges of probenecid-mediated inhibition for SARS-CoV-2 and influenza virus replication [11]. It is known that RSV replication can be affected by several host factors, including importin beta-1, Crm1 [31,32], the actin-binding protein cofilin, caveolin, zinc finger protein ZNF502 [33,34], and serine/threonine protein kinase CK2 [35,36].

Our examination focused on ERK and JNK as we saw no substantial effect on p38 kinase. This finding seemingly contrasts with a published report showing that RSV sequesters phosphorylated p38 into viral inclusion bodies, affecting stress granule assembly during RSV infection [37]. The authors concluded that RSV infection did not appear to affect the accumulation and phosphorylation of p38, but drastically changed its intracellular localization to interfere with the p38 signaling pathway. It is clear that JNKs regulate various cellular processes and can affect virus infection [36,38,39]. Briefly, the MAPK cascade can be initiated by a virus binding to its receptor, which initiates the MAPK signaling cascade via the activation of MAPKK [5]. MAPKK activates MKK4/7 by phosphorylation and MKK4/7 activates the JNKs by phosphorylation [40]. The activated JNKs are translocated to the nucleus [41], activating specific TFs that result in altered gene expression [41]. The TF, c-Jun, has a key role in cell functions like proliferation, apoptosis, survival, tumorigenesis, and tissue morphogenesis [42]. c-Jun is activated by various extracellular signals (e.g., growth factors, cytokines, and extracellular stresses) and enables the production of inflammatory cytokines [38]. Other studies have shown that c-Jun expression can increase the replication of H5N1 influenza virus in human lung epithelial cells, as well as the expression of inflammatory cytokines like TNFα, IFNβ, IL-6, and IL-10 [43]. Similarly, RSV infection induces JNK activity in A549 cells, and inhibiting the JNKs can lower the levels of IL-8 mRNA and protein secretion in the infected cells [44]. c-Jun also mediates the inflammatory responses triggered by these cytokines, forming a potential feedback loop to amplify the inflammatory effects following virus infection; thus, targeting c-Jun is a promising opportunity for therapeutic intervention [45].

The TF, HNF-4, regulates SLC22 transporters including OAT3 [13,46,47]. HNF-4 activity is linked to its phosphorylation by different kinases, including JNKs. When phosphorylated, HNF-4 loses its DNA binding activity, and its nuclear protein levels decrease, but not its mRNA levels [48]. JNK activation has a biphasic nature, with a large increase that lasts for 12 h after stimulation. HNF-4 is a regulator of OAT expression [13]. Interestingly, probenecid not only inhibits JNK phosphorylation, but also reduces reactive oxygen species (ROS) generation by inhibiting the COX-2 and JNK pathways in cultured cells [15]. These pathways are associated with various proinflammatory responses, indicating that probenecid affects both the antiviral and anti-inflammatory responses [49,50].

## 2. Results

### 2.1. MAPK Expression and RSV Infection in Probenecid-Treated A549 Cells

**Figure 1:**
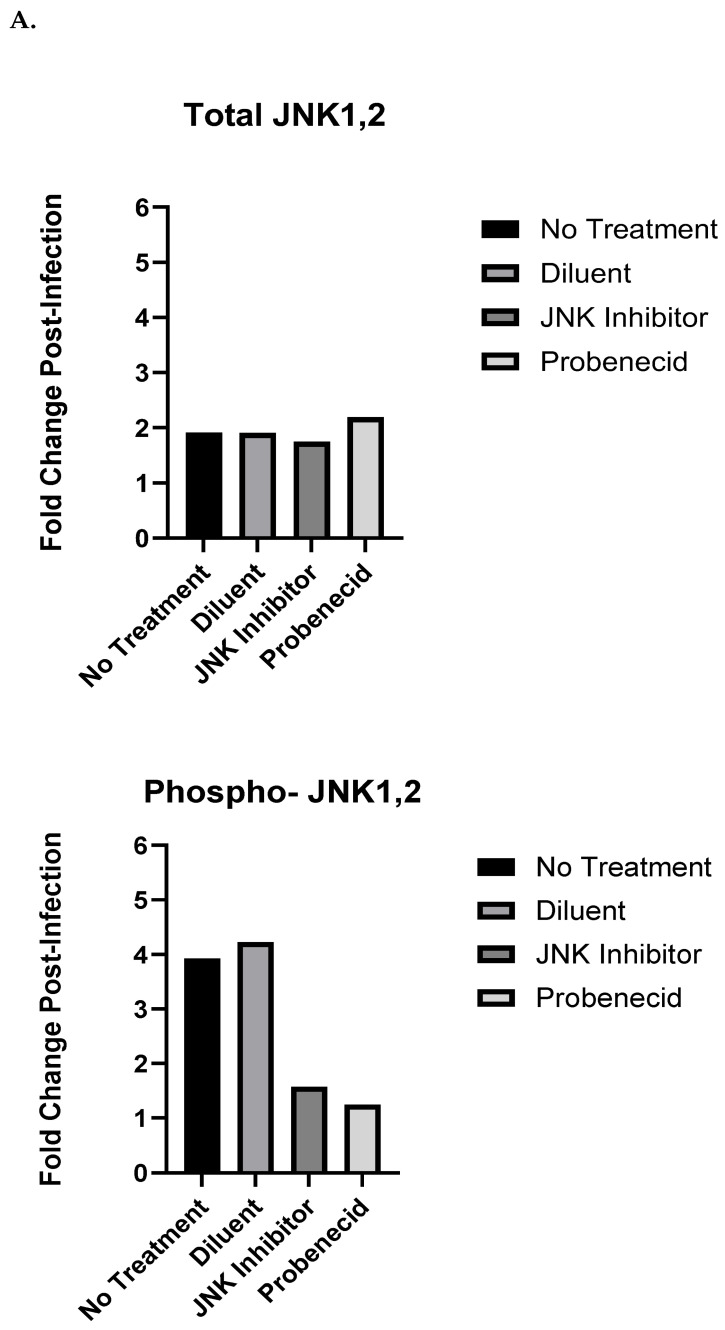

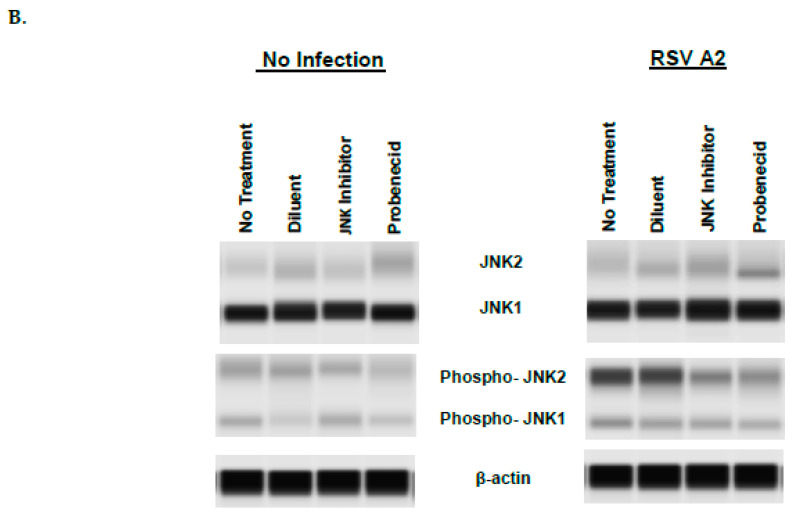
Auto-Western blot analysis of JNK1,2 protein expression and phosphorylation in response to RSV infection of probenecid-treated A549 cells. (**A**) Fold change post-infection of JNK1,2 following treatment. (**B**) Auto-Western blot analysis of JNK1,2 protein expression following treatment. A549 cells were treated with 1 μM probenecid in 0.02% DMSO, or 25 μM SP600125 in 0.02% DMSO, or diluent (0.02% DMSO) for 2 h, or given no treatment. Cells were infected with RSV A2 (MOI = 1.0) for 24 hpi before harvesting, or cells were not infected and cultured for 24 h before harvesting. Culture supernatants were removed, and cells washed and subjected to lysis. Total cell lysates were clarified by centrifugation, and total protein concentration was estimated by BCA protein analysis. Cell lysates were transferred to RayBioTech (Atlanta, GA, USA) for auto-Western blot analysis using their validated antibodies for specific antigen detection. All samples were adjusted to 0.2 mg/mL total protein concentration by RayBioTech prior to auto-Western blot analysis. Experiments were performed with independent replicates for each condition tested. Chemiluminescence values from auto-Western blot readout corresponding to specific band densities from each analyte were normalized to β-actin and mean value calculated for replicate samples. Fold change post-infection was determined by dividing mean normalized band density values corresponding to RSV-infected samples by mean normalized band density values corresponding to uninfected control samples.

RSV infection initiates MAPK signaling in A549 cells [51], so we sought to evaluate the effect of probenecid on MAPK signaling in response to the RSV infection of A549 cells. Other studies have shown that JNK1,2 is an important host factor in RSV production in A549 cells and that peak JNK1,2 activation by phosphorylation occurs at 24 h after RSV infection, correlating with the time required for virus assembly [36]. The JNK family is represented by JNK1 (MAPK8), JNK2 (MAPK9), and JNK3 (MAPK10), which encode for 10 different splice variants, with JNK3 expression restricted to neuronal tissue [52]. ERK1 and ERK2 are 84% identical at the amino acid level, with ERK1 having a 17-amino-acid-long residue insertion at its N-terminus and shares many of the same functions with ERK2 [53]. Sorbitol can activate the JNK, ERK1/2, and p38 MAPK pathways via osmotic stress [54] and is useful as a positive control for the detection of phospho-activated MAPK proteins. The A549 cells treated with 0.5 M sorbitol for 2 h led to strong phospho-JNK1,2, phospho-ERK1/2, and phospho-p38 responses in both the classical and auto-Western blot assays. The phosphorylation of JNK1,2 in the untreated or 0.02% DMSO diluent-treated A549 cells was determined, as was phospho-JNK following infection with RSV A2 (MOI = 1.0) at 24 hpi by auto-Western blots (Figure 1B). The treatment of the RSV-infected A549 cells with a JNK inhibitor (SP600125 in 0.02% DMSO) or 1 μM probenecid (in 0.02% DMSO) for 2 h inhibited the phosphorylation of JNK1,2 (Figure 1B). SP600125 is an ATP-competitive inhibitor of JNK phosphorylation [55]. As expected, the treatment of A549 cells with 25 μM SP600125 for 2 h led to the robust inhibition of phospho-JNK1,2 while the total JNK1,2 was not affected (Figure 1B). Compared to the uninfected, DMSO-treated, or untreated A549 cells, RSV infection resulted in a four-fold increase in phospho-JNK1,2 (Figure 1A). RSV infection resulted in little change in the level of phospho-JNK1,2 proteins at 24 hpi compared to that of the uninfected cells treated with 1 μM probenecid, or SP600125 (Figure 1A). At 24 hpi, the level of total JNK1,2 was increased two-fold for the RSV-infected A549 cells compared to that of the uninfected control cells, while the total JNK1,2 was consistent across all the treatment groups (Figure 1A). To corroborate the auto-Western analysis results, the same samples were examined using classical Western blot to detect the total JNK1,2 and phospho-JNK1,2; the results are consistent with the auto-Western results (Supplementary Figure S2).

**Figure 2:**
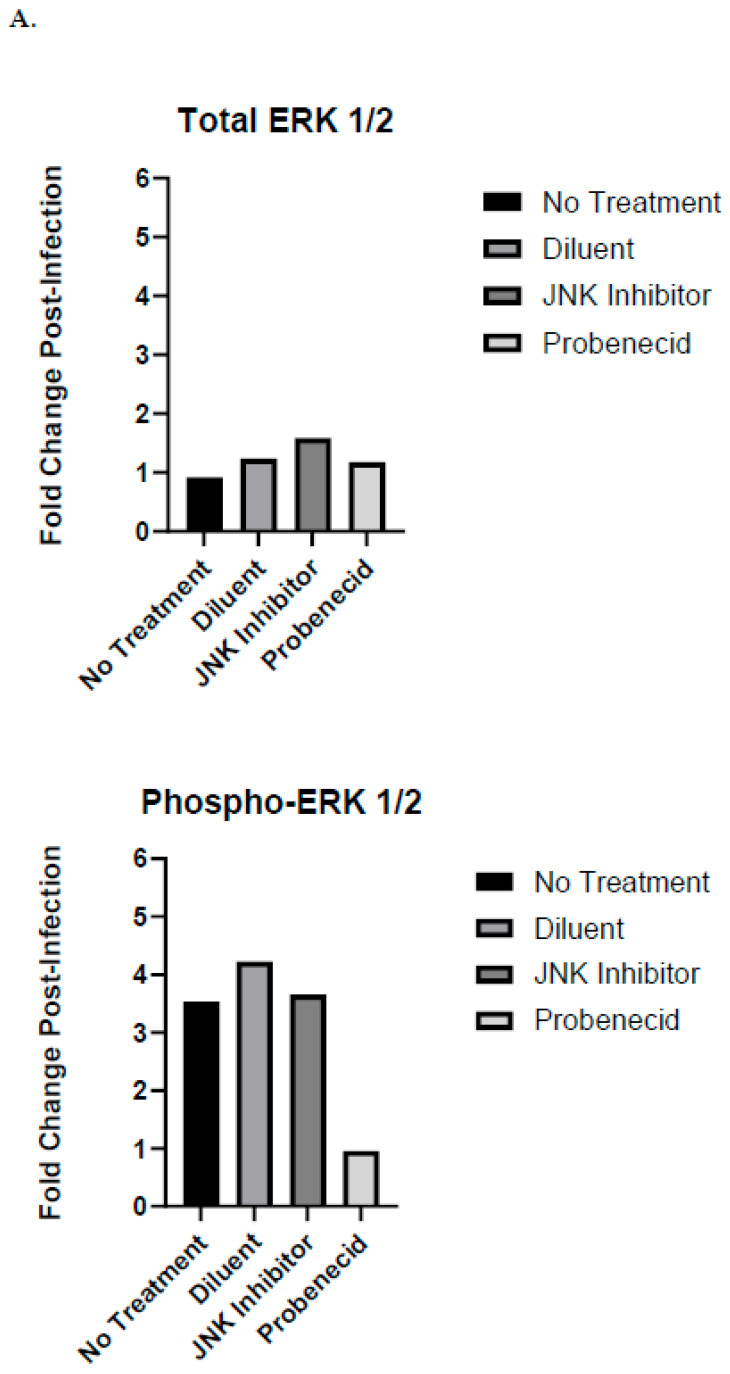

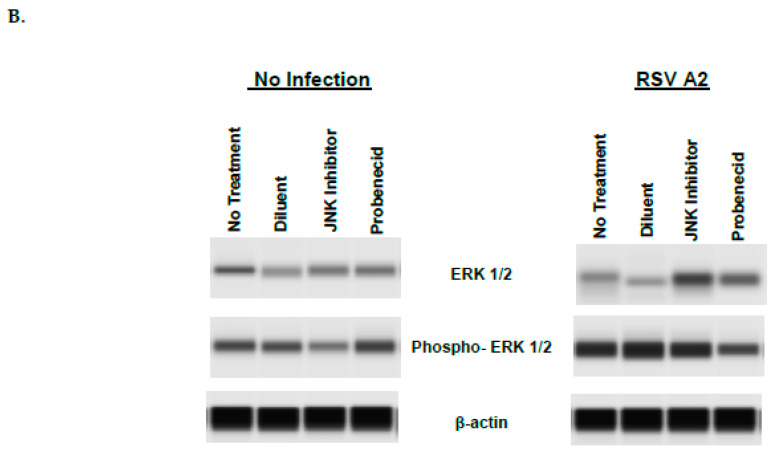
Auto-Western blot analysis of ERK1/2 protein expression and phosphorylation in response to RSV infection of probenecid-treated A549 cells. (**A**) Fold change post-infection of ERK1,2 following treatment. (**B**) Auto-Western blot analysis of ERK1,2 protein expression following treatment. A549 cells were treated with 1 μM probenecid in 0.02% DMSO, or 25 μM SP600125 in 0.02% DMSO, or diluent (0.02% DMSO) for 2 h, or given no treatment. Cells were infected with RSV A2 (MOI = 1.0) for 24 hpi before harvesting, or cells were not infected and cultured for 24 h before harvesting. Culture supernatants were removed, and cells washed and subjected to lysis. Total cell lysates were clarified by centrifugation, and total protein concentration was estimated by BCA protein analysis. Cell lysates were transferred to RayBioTech (Atlanta, GA, USA) for auto-Western analysis using their validated antibodies for specific antigen detection. All samples were adjusted to 0.2 mg/mL total protein concentration by RayBioTech prior to auto-Western blot analysis. Experiments were performed with independent replicates for each condition tested. Chemiluminescence values from auto-Western blot readout corresponding to specific band densities from each analyte were normalized to β-actin and mean value calculated for replicate samples. Fold change post-infection was determined by dividing mean normalized band density values corresponding to RSV-infected samples by mean normalized band density values corresponding to uninfected control samples.

The RSV infection (MOI = 1.0) of the untreated, diluent-treated (0.02% DMSO), or SP600125-treated A549 cells led to ERK1/2 phosphorylation (Figure 2B), while treatment with 1 μM probenecid in 0.02% DMSO inhibited phospho-ERK1/2 (Figure 2B). Compared to the uninfected or untreated A549 cells, or those treated with SP600125, the RSV infection of A549 cells resulted in a four-fold increase in phospho-ERK1/2 (Figure 2A). In contrast, the 1 μM probenecid treatment of the RSV-infected A549 cells resulted in no change in the level of phospho-ERK1/2 at 24 hpi compared to that of the uninfected cells treated with 1 μM probenecid (Figure 2A). The total ERK1/2 levels were slightly increased (1.5-fold) following the RSV infection of the A549 cells treated with SP600125, while the ERK1/2 levels were unchanged in the other treatment groups after RSV infection (Figure 2A). This suggests that the slight increase in phospho-ERK1/2 is not due to changes in the total ERK1/2 levels, but may be linked to cross-talk among the JNK and ERK signaling pathways, as increased ERK1/2 can compensate for the loss in JNK1,2 activity associated with SP600125 treatment (56). Comparing the same samples in the classical Western blot and auto-Western blot results, we found the same levels and expression patterns for the total ERK1/2 and phospho-ERK1/2 (Supplementary Figure S3).

Although phospho-p38 proteins were readily detected in the positive control-treated A549 cells, e.g., 2 h treatments with 0.5 M sorbitol, by both classical and auto-Western blot analyses, phospho-p38 was not detected at 24 hpi in the untreated A549 cells or the A549 cells treated with DMSO diluent, SP600125, 1 μM probenecid, or following RSV infection, but the total p38 proteins were readily detected. Thus, we did not further evaluate the p38 MAPK proteins from the A549 cells.

### 2.2. MAPK Activity and RSV Infection in Probenecid-Treated A549 Cells

**Figure 3:**
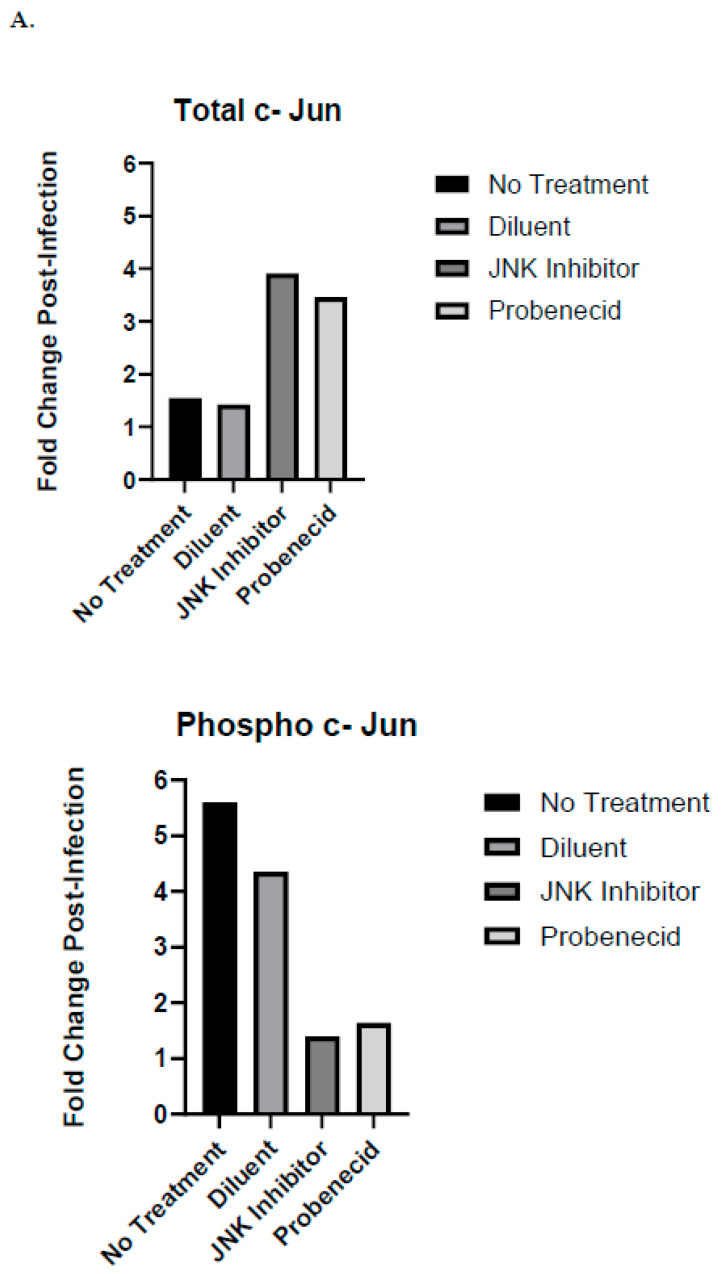

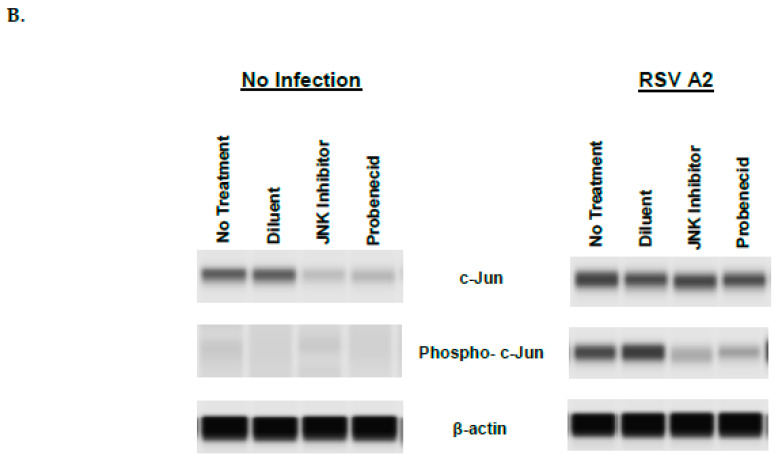
Auto-Western blot analysis of c-Jun protein expression and phosphorylation in response to RSV infection of probenecid-treated A549 cells. (**A**) Fold change post-infection of c-Jun following treatment. (**B**) Auto-Western blot analysis of c-Jun protein expression following treatment. A549 cells were treated with 1 μM probenecid in 0.02% DMSO, or 25 μM SP600125 in 0.02% DMSO, or diluent (0.02% DMSO) for 2 h, or given no treatment. Cells were infected with RSV A2 (MOI = 1.0) for 24 hpi before harvesting, or cells were not infected and cultured for 24 h before harvesting. Culture supernatants were removed, cells washed, and subjected to lysis. Total cell lysates were clarified by centrifugation, and total protein concentration was estimated by BCA protein analysis. Cell lysates were transferred to RayBioTech (Atlanta, GA, USA) for auto-Western analysis using their validated antibodies for specific antigen detection. All samples were adjusted to 0.2 mg/mL total protein concentration by RayBioTech prior to auto-Western blot analysis. Experiments were performed with independent replicates for each condition tested. Chemiluminescence values from auto-Western blot readout corresponding to specific band densities from each analyte were normalized to β-actin and mean value calculated for replicate samples. Fold change post-infection was determined by dividing mean normalized band density values corresponding to RSV-infected samples by mean normalized band density values corresponding to uninfected control samples.

c-Jun comprising a part of the AP-1 transcriptional activating complex is the canonical substrate for JNKs, which mediates c-Jun activation by post-translational phosphorylation. Therefore, c-Jun is one of the terminal phosphoryl group acceptors for the JNK signaling pathway. Other studies have shown that both JNK and ERK1/2, but not the p38 MAPK signaling pathway converge on c-Jun [56]. The untreated A549 cells, or the A549 cells treated with diluent (0.02% DMSO), induced the phosphorylation of c-Jun following infection with RSV A2 (MOI = 1.0) at 24 hpi, as revealed by the auto-Western blot image showing the phospho-c-Jun bands (Figure 3B). In contrast, the phosphorylation of c-Jun was inhibited following RSV infection in the A549 cells treated with SP600125, or 1 μM probenecid in 0.02% DMSO for 2 h (Figure 3B). This is expected as SP600125 is a competitive inhibitor of JNK activity and expected to inhibit the formation of phospho-c-Jun. ERK1/2 can phosphorylate c-Jun, a feature explaining the residual phospho-c-Jun produced in the presence of the JNK inhibitor, SP600125. Compared to the uninfected, diluent-treated, or untreated A549 cells, the RSV infection of the A549 cells led to almost a five-fold increase in phospho-c-Jun proteins (Figure 3A). The level of total c-Jun was increased nearly four-fold following RSV infection at 24 hpi in the A549 cells treated with 1 μM probenecid or treated with SP600125 compared to that of the uninfected cells treated with 1 μM probenecid or treated with SP600125 (Figure 3A). In contrast, the level of total c-Jun proteins was nearly unchanged following RSV infection or treatment with the diluent, or in the untreated A549 cells (Figure 3A). The results show a 2 h treatment with the SP600125 or 1 μM probenecid leads to the depletion of total c-Jun proteins in A549 cells, and following RSV infection, the total c-Jun protein levels are restored at 24 hpi (Figure 3B). c-Jun can potentiate its own promoter activation via AP-1 in a positive feedback circuit [57]. This positive feedback mechanism may help explain the rapid restoration of total c-Jun proteins upon RSV infection. Using the same samples, we confirmed the results in classical and auto-Western blots for c-Jun and phospho-c-Jun after the treatment and RSV infection of the A549 cells (Supplementary Figure S4).

**Figure 4:**
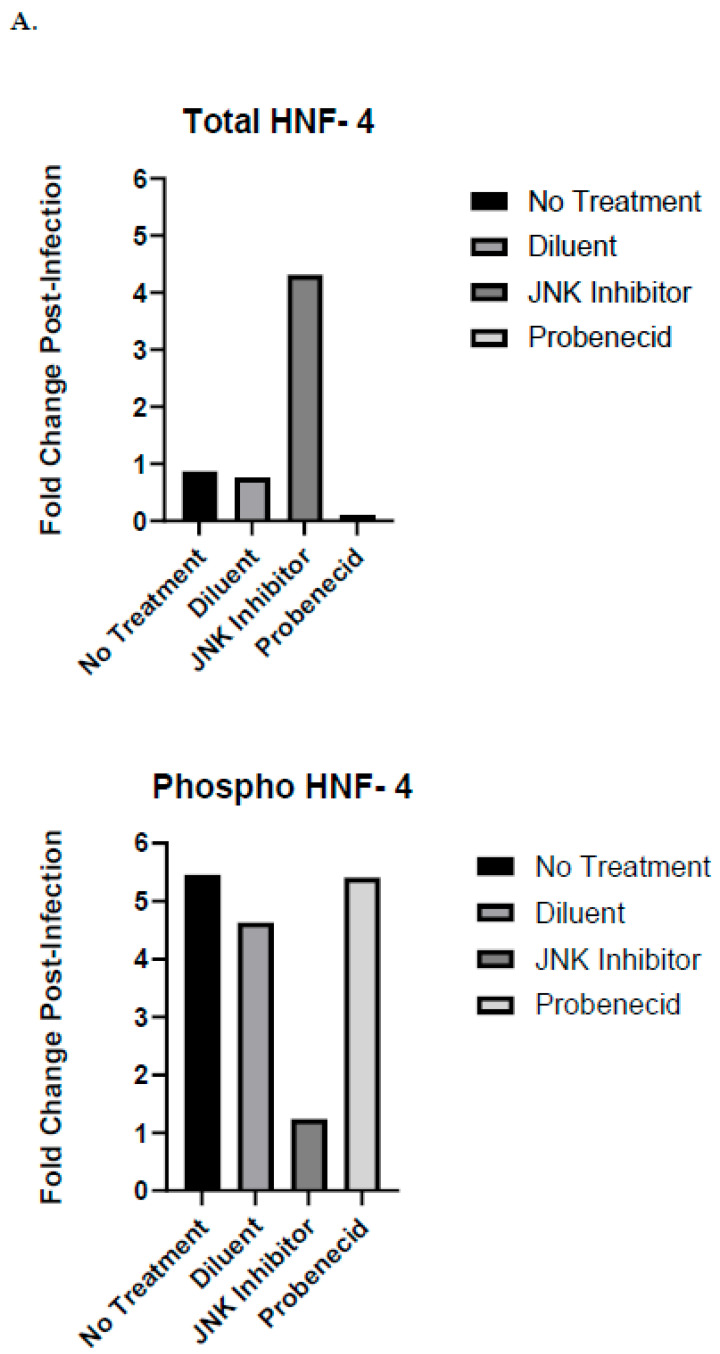

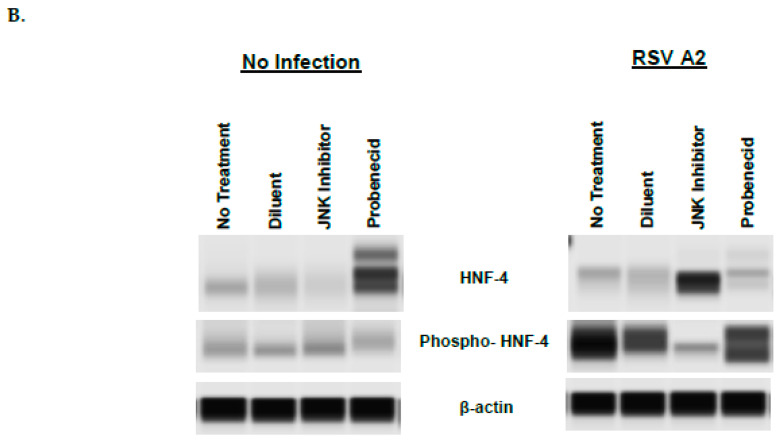
Auto-Western blot analysis of HNF-4 protein expression and phosphorylation in response to RSV infection of probenecid-treated A549 cells. (**A**) Fold change post-infection of HNF-4 following treatment. (**B**) Auto-Western blot analysis of HNF-4 protein expression following treatment. A549 cells were treated with 1 μM probenecid in 0.02% DMSO, or 25 μM SP600125 in 0.02% DMSO, or diluent (0.02% DMSO) for 2 h, or given no treatment. Cells were infected with RSV A2 (MOI = 1.0) for 24 hpi before harvesting, or cells were not infected and cultured for 24 h before harvesting. Culture supernatants were removed, cells washed, and subjected to lysis. Total cell lysates were clarified by centrifugation, and total protein concentration was estimated by BCA protein analysis. Cell lysates were transferred to RayBioTech (Atlanta, GA, USA) for auto-Western blot analysis using their validated antibodies for specific antigen detection. All samples were adjusted to 0.2 mg/mL total protein concentration by RayBioTech prior to auto-Western blot analysis. Experiments were performed with independent replicates for each condition tested. Chemiluminescence values from auto-Western blot readout corresponding to specific band densities from each analyte were normalized to β-actin and mean value calculated for replicate samples. Fold change post-infection was determined by dividing mean normalized band density values corresponding to RSV-infected samples by mean normalized band density values corresponding to uninfected control samples.

HNF-4 is a member of the nuclear receptor superfamily of ligand-dependent transcription factors. Transcription from two different promoters, together with alternative RNA splicing can allow for up to 12 different HNF-4 protein isoforms. Following RSV infection (MOI = 1.0), the untreated or A549 cells treated for 2 h with diluent (0.02% DMSO) or with 1 μM probenecid showed phospho-HNF-4 by auto-Western blotting (Figure 4B). In contrast, the phosphorylation of HNF-4 was inhibited following the 2 h treatment with SP600125 (Figure 4B). Compared to the uninfected, diluent-treated, 1 μM probenecid-treated, or untreated A549 cells, RSV infection resulted in a five-fold increase in phospho-HNF-4 proteins (Figure 4A), while the A549 cells treated with SP600125 had little change in phospho-HNF-4, requiring processing or activation at 24 hpi compared to that of the uninfected cells treated with SP600125 (Figure 4A). These findings are consistent with the activity of SP600125, as HNF-4 protein is a substrate for JNK kinase activity [58]. The levels of total HNF-4 proteins were increased more than four-fold following RSV infection at 24 hpi of the A549 cells treated with SP600125 (Figure 4A). In contrast, following RSV infection, the total HNF-4 proteins was not increased in the untreated or diluent-treated A549 (Figure 4A). The results indicate that the 1 μM probenecid treatment of A549 cells, without RSV infection, increases the total HNF-4 proteins (Figure 4A), and the subsequent RSV infection of these treated cells leads to the nearly complete depletion of detectable total HNF-4 proteins (Figure 4B). These findings are consistent with studies that showed the phosphorylation of HNF-4 protein by JNKs is associated with reduced DNA binding by HNF-4 [59] and that sustained phospho-JNK activation results in the depletion of total HNF-4 proteins. However, low-level and transient JNK activation is associated with the accumulation of non-phosphorylated total HNF-4 proteins. Importantly, probenecid treatment inhibits JNK activation and retards the phosphorylation of HNF-4, thereby maintaining the total HNF-4 protein levels. Using the same set of samples, we corroborated classical and auto-Western analyses to show that the levels and patterns of total HNF-4 proteins and phosphorylated HNF-4 proteins are consistent (Supplementary Figure S5).

Previously siRNA screens revealed a role for OAT3 in influenza virus replication and that siOAT3 reduced the OAT3 mRNA and protein levels in A549 cells [9]. These findings were not revealed for the RSV infection of A549 cells. In this study, the levels of total OAT3 proteins was consistent across all the treatment groups in both the RSV-infected and uninfected cells (Supplementary Figure S1). The preliminary data suggest that OAT3 protein is heavily post-translationally modified by phosphorylation in response to RSV infection. It is unknown what effect this may have on OAT3 activity in the relationship with virus infection. It is possible that the phosphorylation of OAT3 may have a role in the regulation of OAT3 trafficking/recycling for a cellular compartment, such as the ER/Golgi or the plasma membrane, which may affect OAT3 activity. Further studies are required to elucidate the effect of probenecid on the post-translational regulation of OAT3 levels in A549 cells.

## 3. Discussion

Viruses have the ability to take over hosts’ responses to increase their replication. Research has shown that several viruses, such as RSV and others, activate the epidermal growth factor receptor (EGFR), a cell surface receptor used for host cell entry, activating effector proteins and signaling molecules such as MAPKs to suppress the host cells antiviral defense in the airway epithelium [60]. These findings suggest that the virus-induced activation of EGFR is a virulence factor for multiple respiratory viruses. Similarly, inhibiting ERK activation leads to increased RSV-induced IFN expression, thereby exerting a negative effect on the RSV titers. RSV causes the infected cells to undergo apoptosis. However, the activation of ERK delays the virus-induced apoptotic response to infection. When RSV activates EGFR, it leads to an increase in ERK activity, which contributes to both an inflammatory response (IL-8) and the prolonged survival of RSV-infected cells [61]. By reducing ERK activation, probenecid treatment helps re-establish the equilibrium between the antiapoptotic and pro-apoptotic proteins.

JNKs can phosphorylate AP-1 components such as c-Jun. AP-1 regulates the transcription of cytokines and inflammatory enzymes such as COX-2 [62]. Research suggests that viruses use JNK signaling to manipulate their replication. JNK activation may favor viral replication, while inhibiting JNK activation may lead to reduced viral replication, suggesting that JNK can regulate viral replication at various stages during virus replication. The JNKs are activated when the virus binds to its receptor, as ligand–receptor interaction is sufficient to activate the JNKs. For example, the interaction between UV-inactivated dengue virus and its receptor triggers early JNK phosphorylation and activation during entry and infection [63]. Research has shown that cells treated with selective JNK inhibitors exhibited the pharmacological inhibition of JNK activation, resulting in the reduction of viral RNA synthesis, the viral protein expression level, and progeny release. The JNK signaling pathway regulates various cellular processes, helping to maintain a balance between cell survival and death in response to stress and infection. For example, JNK signaling directs the phosphorylation and repression of HNF-4, which mediates homeostasis and cell survival [64]. The inflammatory cytokine IL-1β activates JNK signaling, which, in turn, reduces the levels of total HNF-4 protein and inhibits transcription of HNF-4 target genes. Probenecid impairs the phosphorylation of JNKs, thereby reducing the expression of phospho-c-jun protein, and probenecid reverses the repression of HNF-4 protein, which is a separate JNK substrate that is repressed by JNK signaling. Blocking JNK signaling not only prevents virus replication, but also has a crucial role in autophagy. Viruses exploit autophagy to replicate, and this leads to endoplasmic reticulum (ER) stress during virion release. For example, RSV infection has been shown to trigger ER stress in primary human tracheobronchial epithelial cells and A549 cells by producing large amounts of viral glycoproteins [65]. These proteins likely compete with the host proteins for processing, leading to an increased burden on ER/Golgi trafficking.

## 4. Materials and Methods

### 4.1. Cell Culture and Viral Infection

A549 human lung epithelial cells (ATCC CCL-185) were propagated in DMEM (Gibco, Waltham, MA, USA) + 10% heat-inactivated FBS (Hyclone, Logan, UT, USA) at 5% CO_2_ at 37 °C. RSV A2 (ATCC VR-1540) was propagated and quantified on A549 cells, and then stored at −80 °C. RSV titers were determined using a methylcellulose plaque assay. A549 cells were infected with RSV A2 at MOI = 1 in incomplete DMEM (Gibco) for 1 h. The cells were changed into fresh complete DMEM and cultured for an additional 24 h at 37 °C.

### 4.2. Probenecid Mediated Inhibition of RSV Replication

A working stock of probenecid (Sigma-Aldrich, St. Louis, MO, USA) or JNK inhibitors (SP600125) (Sigma-Aldrich) was dissolved in DMSO (Sigma-Aldrich), and dilutions of the working stock were resuspended in PBS (Gibco). Cellular toxicity was determined using a ToxiLight Bioassay (Lonza, Basel, Switzerland). A549 cells were plated overnight at 10^5^ cells/well in 6-well flat-bottom plates (Corning Costar, Corning, NY, USA). Cells were treated for 2 h with probenecid at 1 µM, or SP600125 at 25 µM, or diluent only (0.02% DMSO) prior to infection. After treatment, the media were removed, and the cells were infected with RSV A2 at MOI = 1. At 24 h post-infection, the plates containing the cells were placed on ice, the supernatants were removed, and total protein was extracted from the cells.

### 4.3. Cell Lysis and Western Blot Analysis

Culture supernatants were removed, and the cells were washed once with ice cold PBS, and then subjected to lysis in RIPA buffer (1% sodium deoxycholate, 0.5% Triton X-100, 50 mM Tris-HCL pH 7.5, 1 mM EDTA, 1mM PMSF), ‘complete’ protease inhibitor tablets (MilliporeSigma, Burlington, MA, USA), and phosphatase inhibitor tablets (Thermo Fisher, Waltham, MA, USA). Total cell lysates were kept on ice and incubated for 5 min after mixing. Lysates were clarified by centrifugation, and the total protein concentration was estimated by BCA protein analysis. Lysates were aliquoted and stored at −80 °C until they were transferred to RayBiotech (Atlanta, GA, USA) for auto-Western analysis. RayBiotech’s auto-Western service was used because it combines a state-of-the-art capillary immunoprobing system with a pre-validated antibody library and is a highly accurate and precise capillary immunoassay method. The results were generated in electropherogram format, with each peak displayed numerically, and then electropherogram peaks were digitally rendered as a virtual blot. This high-throughput system can achieve nanogram-to-picogram sensitivity with only 5 µL of starting material.

For classical Western blots, equal amounts of cell lysates (30 μg) were separated on 4–20% gradient SDS-PAGE gels (BioRad, Hercules, CA, USA), and then transferred to nitrocellulose membrane (BioRad, Hercules, CA, USA) for immunoprobing. Membranes were blocked in Tris-buffered pH = 7.5 saline solution containing 0.05% Tween-20 (MilliporeSigma, Burlington, MA, USA), (TBS-T) and 5% BSA for 1 h at room temperature, and then incubated with the primary antibody in TBS-T overnight at 4 °C. Specifically, anti-phospho-JNK1/JNK2/JNK3 (Thr183, Thr221) recombinant rabbit monoclonal antibody (mAb) (Thermo Fisher, Waltham, MA, USA), anti-JNK1/JNK2/JNK3 recombinant rabbit mAb (Thermo Fisher, Waltham, MA, USA), anti-HNF-4 polyclonal antibody (pAb) (Thermo Fisher, Waltham, MA, USA), anti-phospho-HNF4 (Ser304) pAb (Thermo Fisher, Waltham, MA, USA), anti-SLC22A8 (OAT3) pAb (Thermo Fisher, Waltham, MA, USA), anti-phospho-SLC22A8 (Ser4) pAb (Thermo Fisher, Waltham, MA, USA), anti-phospho-c-Jun (Ser73) pAb (Thermo Fisher, Waltham, MA, USA), anti-β-Actin pAb (Thermo Fisher, Waltham, MA, USA), c-Jun (60A8) rabbit mAb, phospho-c-Jun (Ser63) (54B3) rabbit mAb (Cell Signaling Technologies, Danvers, MA, USA), p44/42 MAP kinase (137F5) rabbit mAb, and Phospho-p44/42 MAPK (ERK1/2) (Cell Signaling Technologies, Danvers, MA, USA) was used. After three washes in TBS-T, the membranes were incubated with mouse anti-rabbit IgG (H + L) cross-adsorbed Secondary Antibody, HRP conjugate (Thermo Fisher, Waltham, MA, USA), in TBS-T for 2 h at room temperature. The membranes were washed three times with TBS-T, and the signal was developed with ECL (Thermo Fisher, Waltham, MA, USA) chemiluminescent substrate. Immunoblot images were obtained using a FluorChemE instrument (Protein Simple, Minneapolis, MN, USA).

### 4.4. Chemicals

The ATP-competitive JNK inhibitor (SP600125), sorbitol, an osmotic stress-inducing JNK activator, and probenecid were obtained from MilliporeSigma, Burlington, MA, USA.

### 4.5. Statistical Analysis

This study used independent replicate samples for all Western blots. The fold-change in MAPK protein levels in response to the RSV infection of A549 cells was determined by dividing the signal from the specific MAPK proteins from the infected and uninfected cells by the signal from the β-actin proteins in the same samples. From the mean values obtained from the replicate samples, the mean normalized MAPK protein signal from the RSV-infected cells was divided by the mean normalized MAPK protein signal from uninfected cells, resulting in the ratio presented in the figures. Where possible, statistics were determined using GraphPad Prism, version 9.

## 5. Conclusions

Our research has shown that probenecid treatment can inhibit RSV replication in A549 cells by preventing JNK signaling. Probenecid is broadly antiviral and effective against many variants and strains of SARS-CoV-2, influenza virus, and RSV [9,10,11,12,16,30]. Importantly, in a Phase two clinical study, probenecid was more effective in treating mild-to-moderate COVID-19 in non-hospitalized patients compared to that the treatment of controls [16]. These findings suggest that probenecid’s pan-antiviral activity is linked to its targeting of JNK signaling. JNK activation is crucial for virus replication, and inhibiting JNK activation can reduce viral replication in human viruses like dengue, rotavirus, and influenza, as well as veterinary viruses like infectious bursal disease virus (IBDV), porcine epidemic diarrhea virus (PEDV), and porcine reproductive and respiratory syndrome virus (PRRSV) [39,66]. Host factors, such as OAT3, are essential for some viral replication pathways such as RSV and could serve as pharmacological targets as well in the development of antiviral therapeutics.

## Data Availability

All the data are contained within the manuscript. The data supporting reported results can be found archived datasets generated in the Tripp laboratory at UGA.

## Supplementary Materials

The supporting information can be downloaded at https://www.mdpi.com/article/10.3390/ijms252212452/s1.
