# Supplementary material for: Probenecid Inhibits Extracellular Signal-Regulated Kinase and c-Jun N-Terminal Kinase Mitogen-Activated Protein Kinase Pathways in Regulating Respiratory Syncytial Virus Response
Source: Int J Mol Sci. 2024 Nov 20;25(22):12452. doi: 10.3390/ijms252212452 (PMC11594929; doi:10.3390/ijms252212452)
Supplement: Supplementary file 1 [file ijms-25-12452-s001.zip › ijms-3277084-supplementary.pdf]

Supplementary Figure S1. Auto-Western blot analysis of OAT3 protein expression in response to RSV infection of probenecid-treated A549 cells.

A.

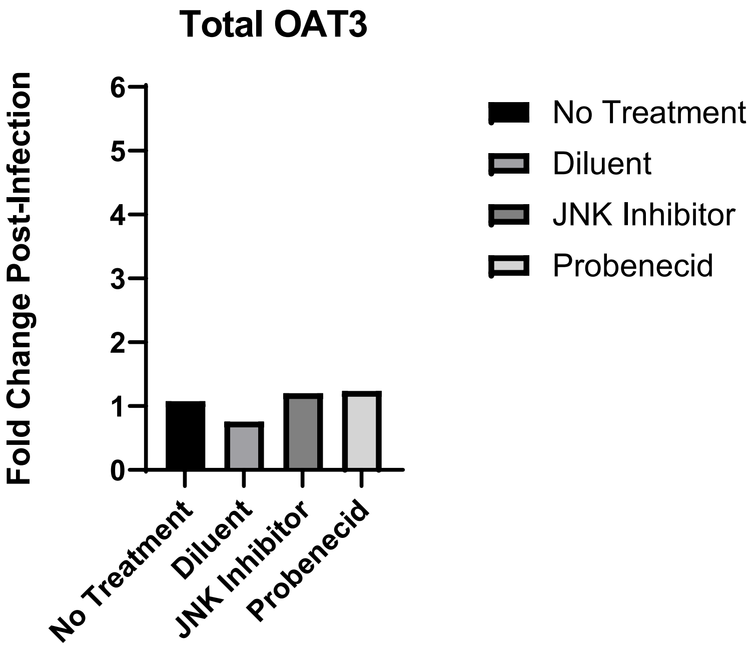

B.

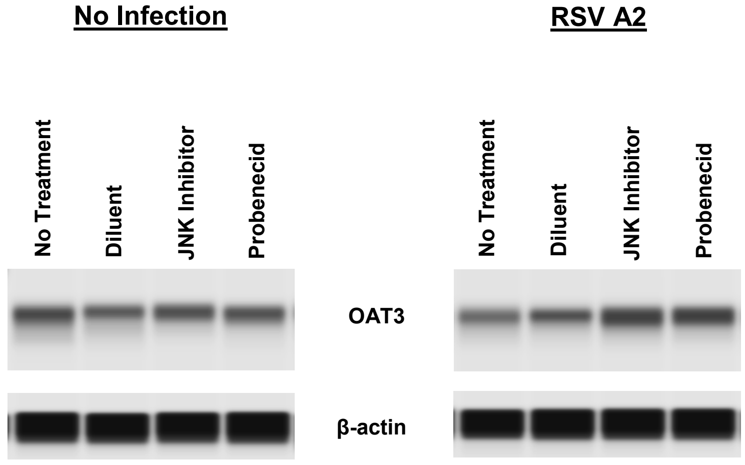

Supplementary Figure S1A,B legend. A549 cells were treated with 1 μM probenecid in 0.02% DMSO, or 25 μM SP600125 in 0.02% DMSO, or diluent (0.02% DMSO) for 2 h, or no treatment. Cells were infected with RSV A2 (MOI=1.0) for 24 hpi before harvesting, or cells were not infected and cultured for 24 h before harvesting. Culture supernatants were removed, the cells washed, and subjected to lysis. Total cell lysates were clarified by centrifugation, and the total protein concentration was estimated by BCA protein analysis. Cell lysates were transferred to RayBioTech (Atlanta, GA) for auto-Western analysis using their validated antibodies for specific antigen detection. All samples were adjusted to 0.2 mg/ml total protein concentration by RayBioTech prior to auto-Western blot analysis. The experiment was performed with independent replicates for each condition tested. Chemiluminescence values from the auto-Western blot readout corresponding to specific band densities from each analyte were normalized to β-actin and the mean value calculated for replicate samples. Fold change post-infection was determined by dividing the mean normalized band density values corresponding to the RSV infected samples by the mean normalized band density values corresponding to the No Infection control samples.

Supplementary Figure S2. Classical Western blots of JNK1,2 expression and phosphorylation in response to RSV infection of probenecid-treated A549 cells.

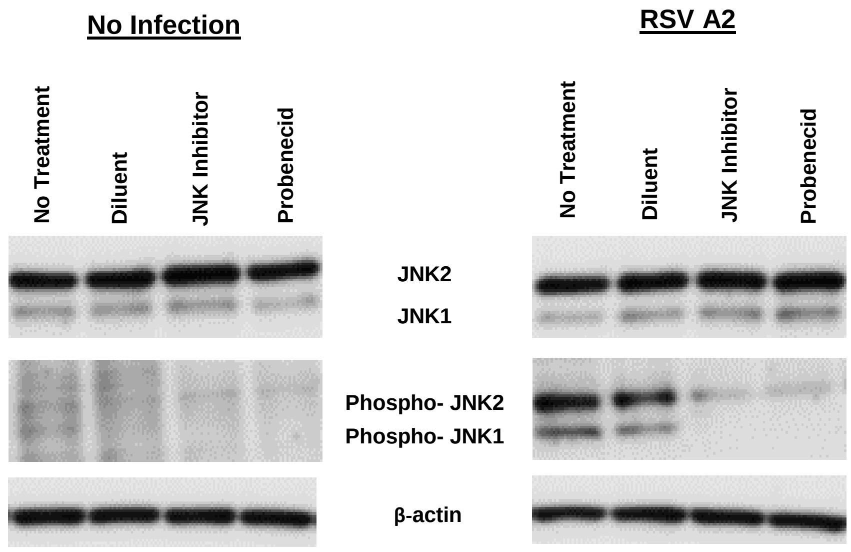

Supplementary Figure S2 legend. A549 cells were treated with 1 μM probenecid in 0.02% DMSO, or 25 μM SP600125 in 0.02% DMSO, or diluent (0.02% DMSO) for 2 h, or no treatment. Cells were infected with RSV A2 (MOI=1.0) for 24 hpi before harvesting, or cells were not infected and cultured for 24 h. Culture supernatants were removed and cells were washed once with ice-cold PBS and then subjected to lysis in RIPA buffer (1% sodium deoxycholate, 0.5% Triton X-100, 50 mM Tris-HCL pH 7.5, 1 mM EDTA, 1 mM PMSF, 'complete' protease inhibitor tabs (MilliporeSigma, Burlington, MA), and phosphatase inhibitor minitabs (Thermo Fisher, Waltham, MA). Total cell lysates were kept on ice and incubated for 5 min after mixing. Lysates were clarified by centrifugation, and the total protein concentration was estimated by BCA protein analysis. Total cell lysates, 30 μg protein per lane, were used for Western blotting on 4-20% gradient SDS-PAGE gels (BioRad, Hercules, CA) then transferred to nitrocellulose membrane (BioRad, Hercules, CA) for immunoprobing. Membranes were probed with antibodies (anti-phospho-JNK1/JNK2/JNK3 (Thr183, Thr221) recombinant rabbit monoclonal antibody (mAb) (Thermo Fisher, Waltham, MA), anti-JNK1/JNK2/ JNK3 recombinant rabbit mAb (Thermo Fisher, Waltham, MA), and mouse anti-rabbit

IgG (H+L) cross-adsorbed Secondary Antibody, HRP conjugate (Thermo Fisher, Waltham, MA) and the signal developed with a chemiluminescent substrate (ECL; Thermo Fisher, Waltham, MA). Immunoblot images were obtained using the FluorChemE instrument (Protein Simple, Minneapolis, MN).

Supplementary Figure S3. Classical Western blots of ERK1/2 protein expression and phosphorylation in response to RSV infection of probenecid-treated A549 cells.

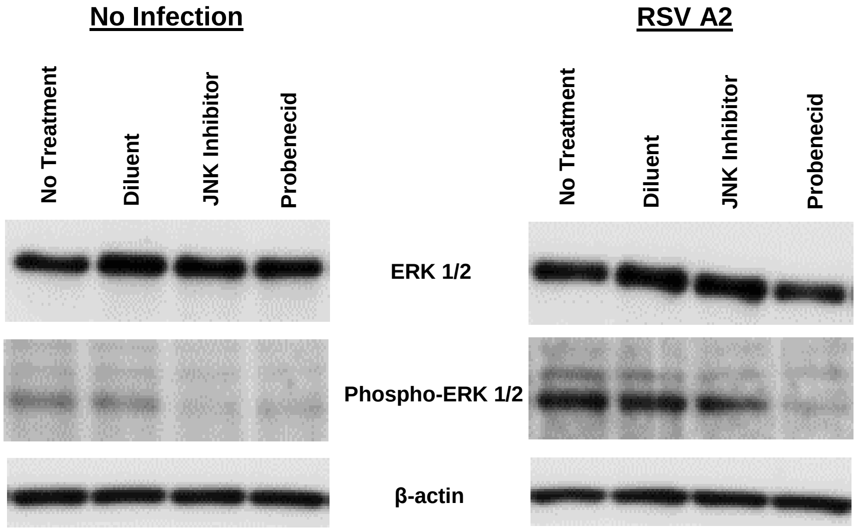

Supplementary Figure S3 legend. A549 cells were treated with 1 μM probenecid in 0.02% DMSO, or 25 μM SP600125 in 0.02% DMSO, or diluent (0.02% DMSO) for 2 h, or no treatment. Cells were infected with RSV A2 (MOI=1.0) for 24 hpi before harvesting, or cells were not infected and cultured for 24 h. Culture supernatants were removed and cells were washed once with ice-cold PBS and then subjected to lysis in RIPA buffer (1% sodium deoxycholate, 0.5% Triton X-100, 50 mM Tris-HCL pH 7.5, 1 mM EDTA, 1 mM PMSF, 'complete' protease inhibitor tabs (MilliporeSigma, Burlington, MA), and phosphatase inhibitor minitabs (Thermo Fisher, Waltham, MA). Total cell lysates were kept on ice and incubated for 5 min after mixing. Lysates were clarified by centrifugation, and the total protein concentration was estimated by BCA protein analysis. Total cell lysates 30 μg protein per lane were used for Western blotting on 4 - 20% gradient SDS-PAGE gels (BioRad, Hercules, CA) then transferred to nitrocellulose membrane (BioRad, Hercules, CA) for immunoprobing. Membranes were probed with antibodies p44/42 MAP kinase (137F5) rabbit mAb and Phospho-p44/42 MAPK (ERK1/2) (Cell Signaling Technologies, Danvers, MA) and mouse anti-rabbit IgG (H+L) cross-adsorbed secondary

antibody, HRP conjugate (Thermo Fisher, Waltham, MA). and the signal developed with a chemiluminescent substrate (ECL; Thermo Fisher, Waltham, MA). Immunoblot images were obtained using the FluorChemE instrument (Protein Simple, Minneapolis, MN).

Supplementary Figure S4. Classical Western blots of c-Jun protein expression and phosphorylation in response to RSV infection of probenecid-treated A549 cells.

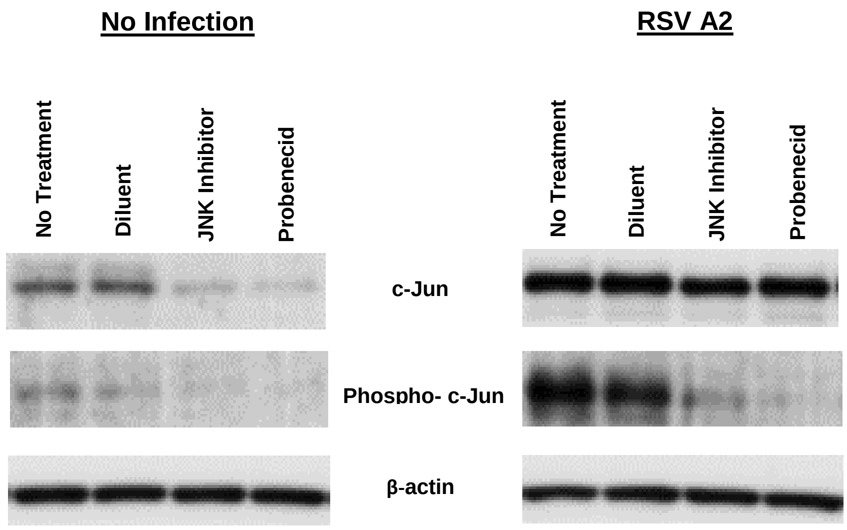

Supplementary Figure S4 legend. A549 cells were treated with 1 µM probenecid in 0.02% DMSO, or 25 µM SP600125 in 0.02% DMSO, or diluent (0.02% DMSO) for 2 h, or no treatment. Cells were infected with RSV A2 (MOI=1.0) for 24 hpi before harvesting, or cells were not infected and cultured for 24 h. Culture supernatants were removed and cells were washed once with ice-cold PBS and then subjected to lysis in RIPA buffer (1% sodium deoxycholate, 0.5% Triton X-100, 50 mM Tris-HCL pH 7.5, 1 mM EDTA, 1 mM PMSF, 'complete' protease inhibitor tabs (MilliporeSigma, Burlington, MA), and phosphatase inhibitor minitabs (Thermo Fisher, Waltham, MA). Total cell lysates were kept on ice and incubated for 5 min after mixing. Lysates were clarified by centrifugation, and the total protein concentration was estimated by BCA protein analysis. Total cell lysates, 30 µg protein per lane, were used for Western blotting on 4 - 20% gradient SDS-PAGE gels (BioRad, Hercules, CA) then transferred to nitrocellulose membrane (BioRad, Hercules, CA) for immunoprobing. Membranes were probed with antibodies c-Jun (60A8) rabbit mAb and phospho-c-Jun (Ser63) (54B3) rabbit mAb (Cell Signaling Technologies, Danvers, MA) and mouse anti-rabbit IgG (H+L) cross-adsorbed Secondary Antibody,

HRP conjugate (Thermo Fisher, Waltham, MA) and the signal developed with a chemiluminescent substrate (ECL; Thermo Fisher, Waltham, MA). Immunoblot images were obtained using the FluorChemE instrument (Protein Simple, Minneapolis, MN).

Supplementary Figure S5. Classical Western blots of HNF-4 protein expression and phosphorylation in response to RSV infection of probenecid-treated A549 cells.

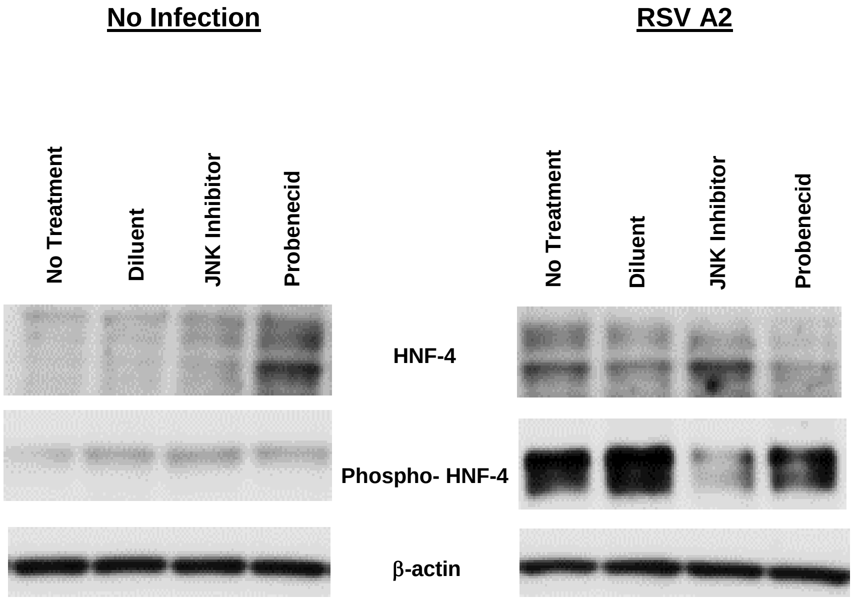

Supplementary Figure S5 legend. A549 cells were treated with 1 μM probenecid in 0.02% DMSO, or 25 μM SP600125 in 0.02% DMSO, or diluent (0.02% DMSO) for 2 h, or no treatment. Cells were infected with RSV A2 (MOI=1.0) for 24 hpi before harvesting, or cells were not infected and cultured for 24 h. Culture supernatants were removed and cells were washed once with ice-cold PBS and then subjected to lysis in RIPA buffer (1% sodium deoxycholate, 0.5% Triton X-100, 50 mM Tris-HCL pH 7.5, 1 mM EDTA, 1 mM PMSF, 'complete' protease inhibitor tabs (MilliporeSigma, Burlington, MA), and phosphatase inhibitor minitabs (Thermo Fisher, Waltham, MA). Total cell lysates were kept on ice and incubated for 5 min after mixing. Lysates were clarified by centrifugation, and the total protein concentration was estimated by BCA protein analysis. Total cell lysates 30 μg protein per lane were used for Western blotting on 4 - 20% gradient SDS-PAGE gels (BioRad, Hercules, CA) then transferred to nitrocellulose membrane (BioRad, Hercules, CA) for immunoprobing. Membranes were probed with

antibodies anti-HNF4 polyclonal antibody (pAb) (Thermo Fisher, Waltham, MA), anti-phospho-HNF4 (Ser304) pAb (Thermo Fisher, Waltham, MA), and mouse anti-rabbit IgG (H+L) cross-adsorbed secondary antibody, HRP conjugate (Thermo Fisher, Waltham,MA) and the signal developed with a chemiluminescent substrate (ECL; Thermo Fisher, Waltham, MA). Immunoblot images were obtained using the FluorChemE instrument (Protein Simple, Minneapolis, MN).
